# Microbiota gut-brain axis: implications for pediatric-onset leukodystrophies

**DOI:** 10.3389/fnut.2024.1417981

**Published:** 2024-07-12

**Authors:** Ylenia Vaia, Fabio Bruschi, Veronica Maria Tagi, Martina Tosi, Chiara Montanari, Gianvincenzo Zuccotti, Davide Tonduti, Elvira Verduci

**Affiliations:** ^1^C.O.A.L.A. (Center for Diagnosis and Treatment of Leukodystrophies), Unit of Pediatric Neurology, V. Buzzi Children’s Hospital, Milan, Italy; ^2^Department of Biomedical and Clinical Sciences, University of Milan, Milan, Italy; ^3^Department of Pediatrics, V. Buzzi Children's Hospital, Milan, Italy; ^4^Department of Health Sciences, University of Milan, Milan, Italy; ^5^Metabolic Diseases Unit, Department of Pediatrics, V. Buzzi Children’s Hospital, University of Milan, Milan, Italy

**Keywords:** neurodegenerative disorders, leukodystrophies, gut-brain axis, dysbiosis, gut microbiota

## Abstract

Neurodegenerative disorders are a group of diseases characterized by progressive degeneration of the nervous system, leading to a gradual loss of previously acquired motor, sensory and/or cognitive functions. Leukodystrophies are amongst the most frequent childhood-onset neurodegenerative diseases and primarily affect the white matter of the brain, often resulting in neuro-motor disability. Notably, gastrointestinal (GI) symptoms and complications, such as gastroesophageal reflux disease (GERD) and dysphagia, significantly impact patients’ quality of life, highlighting the need for comprehensive management strategies. Gut dysbiosis, characterized by microbial imbalance, has been implicated in various GI disorders and neurodegenerative diseases. This narrative review explores the intricate relationship between GI symptoms, Gut Microbiota (GM), and neurodegeneration. Emerging evidence underscores the profound influence of GM on neurological functions via the microbiota gut-brain axis. Animal models have demonstrated alterations in GM composition associated with neuroinflammation and neurodegeneration. Our single-centre experience reveals a high prevalence of GI symptoms in leukodystrophy population, emphasizing the importance of gastroenterological assessment and nutritional intervention in affected children. The bidirectional relationship between GI disorders and neurodegeneration suggests a potential role of gut dysbiosis in disease progression. Prospective studies investigating the GM in leukodystrophies are essential to understand the role of gut-brain axis dysfunction in disease progression and identify novel therapeutic targets. In conclusion, elucidating the interplay between GI disorders, GM, and neurodegeneration holds promise for precision treatments aimed at improving patient outcomes and quality of life.

## 1 Introduction

Neurodegenerative disorders are a group of diseases characterized by progressive degeneration of the structures composing the central and/or peripheral nervous system, leading to a gradual loss of previously acquired motor, sensory and/or cognitive functions. Most common neurodegenerative disorders are typical of adulthood, such as Parkinson disease, and some forms of dementia (i.e., Alzheimer disease, Lewy body dementia, etc), while they are rarer entities in children. Childhood-onset neurodegenerative diseases pose unique challenges for paediatric neurologists as they may show overlapping symptoms with other neurological conditions; loss of motor skills, cognitive deterioration, feeding difficulties, and vision and/or hearing impairment are common features of different neurological diseases, and often the same disease may display different clinical presentations. An algorithm for the management of children with suspected neurodegenerative disorders and a classification system for these conditions, based on the prominently involved structures (i.e., disorders with prominent involvement of cerebral grey matter, leukoencephalopathies, etc.) has been developed (1). Leukodystrophies make up a significant proportion of pediatric-onset neurodegenerative conditions (2).

## 2 Leukodystrophies

Leukodystrophies are a heterogeneous group of rare genetic neurodegenerative disorders that affect children, primarily involving the white matter of the brain (3). Leukodystrophies can be classified upon the white matter component primarily involved and can be distinguished in myelin disorders, astrocytopathies, leukoaxonopathies, microgliopathies and leukovasculopathies (4). According to the neuroradiological patterns we can define hypomyelinating forms, characterized by an arrest of the formation and maturation of myelin, and other disorders, mainly represented by demyelinating forms, characterized by progressive degeneration of the white matter (4). A consensus has been built among a panel of leukodystrophy specialists regarding the definition of the term leukodystrophy. The panel comprehensively identified disorders that align with the established definition, creating a list of known leukodystrophies. Additionally, the group introduced the term ‘genetic leukoencephalopathy (gLE)’ to describe hereditary disorders causing white matter abnormalities that do not strictly meet the criteria for leukodystrophies (3). Although aetiology varies across conditions, an alteration in metabolic/cytohistological processes commonly represents the disease cause, and neuroinflammation might boost disease progression (5).

From a clinical point of view, the involvement of white matter tracts almost always impacts motor abilities, leading to various degrees of motor impairment usually related to pyramidal signs and/or ataxia. Other variable symptoms may include extrapyramidal movement disorders (mainly dystonia), seizures, delays or changes in cognitive development over time, visual and auditory impairment, extra neurological signs and symptoms depending on the specific disorder (6).

The hereditary nature of leukodystrophies, combined with their monogenic origin, has facilitated the development of some animal models. These are extensively employed in biomedical research because of their potential to replicate some aspects of human diseases, thus enabling an in-depth investigation of pathophysiological processes. Rutherford and Hamilton (7) provided a review of animal models of some of the most common leukodystrophies, such as X-linked adrenoleukodystrophy (X-ALD), metachromatic leukodystrophy (MLD), Krabbe’s disease (KD), Alexander disease (AxD), and Aicardi-Goutières syndrome (AGS) and highlighted their usefulness in identifying new cellular drivers and their potential target for new therapeutic strategies (7). Though, despite their significant contribution in understanding leukodystrophies pathogenesis, reliability on disease progression and response to experimental treatments remain scarce, largely due to the lack of animal models that fully and adequately mimic human disease, particularly white matter pathology. The translational gap necessitates the use of complementary methodologies, such as computational models, human cell-based systems, and clinical studies, to enhance the relevance and applicability of preclinical findings to human health and disease.

## 3 GI disorders in leukodystrophies and nutritional interventions: insights from literature

Leukodystrophies often entail life-challenging gastrointestinal (GI) complications, with gastroesophageal reflux disease (GERD), recurrent vomiting, and bowel dysfunction being the most frequent concerns, often affecting appetite and growth patterns (8). In addition, dysphagia is a very frequent, disabling and sometimes fatal symptom. It is linked with the risk of malnutrition and exposes patients to the dangers of aspiration pneumonia or airway obstruction (9). It recognizes a multifactorial origin (neurogenic, postural, iatrogenic, upper gastrointestinal tract dysfunction) and can cause dehydration, chronic malnutrition, failure to thrive, and depletion of essential nutrients (10). Anorexia has also been described in leukodystrophies (11).

GI disorders represent a challenging problem and significantly increase the burden of disease in these patients. They can primarily be related to disease pathogenesis, such as in AxD (12), or can be a consequence of severe neurological disability, like what is usually observed in cerebral palsy (13). Sometimes, an earlier onset of GI complications has been related to an earlier disease onset, as described in MLD (14). A proper nutritional assessment and intervention can ameliorate the nutritional status of children with leukodystrophies (10). Given the extreme phenotypic variability, nutritional intervention must be directed to meet the individual patient’s needs, usually targeting the specific symptoms and complications to improve patients’ quality of life. Specific dietetic approaches have been explored as therapeutic intervention for some leukodystrophies. Ketogenic diet has shown to promote myelination in mouse models of Pelizaeus Merzbacher Disease (15), and has been administered in isolated cases of leukodystrophy (16, 17). Additionally, it is well known that dietary intervention plays a significant role in X-ALD, with a diet that is primarily characterized by the restriction of Very Long Chain Fatty Acids (VLCFA) and the augmentation of peroxisomal beta-oxidation through the administration of a combination of antioxidant compounds, conjugated linoleic acid (CLA), and Lorenzo’s oil (LO) [a 4:1 mixture of glyceryl trioleate (GTO) (C18:1 n-9) and glyceryl trierucate (GTE) (C22:1 n-9)], conjugated linoleic acid (CLA), and antioxidants (18).

## 4 GI disorders in leukodystrophies: an Italian single center experience

Out of 175 patients referred to our Centre for Diagnosis and Care of Leukodystrophies and Associated Conditions (C.O.A.L.A.) at *V. Buzzi* Children’s Hospital in Milan, Italy, who were diagnosed with either a leukodystrophy or a genetic leukoencephalopathy (Supplemenatry Table S1), data on gastrointestinal symptoms were available for 133 (76%). More than half of our cohort (75 patients, 56.4%) had GI manifestations. 35 individuals (26.3% of the cohort) reported one GI symptom, 15 (11.3%) were diagnosed with 2 gastrointestinal symptoms, while 3 or more manifestations were observed in 25 individuals (18.8%) (Table 1).

**Table 1:** Distribution of GI symptoms in the population affected by leukodystrophies or genetic leukoencephalopathies referred to the centre for diagnosis and care of leukodystrophies and associated conditions (C.O.A.L.A.) at V. Buzzi Children’s Hospital in Milan, Italy.

	N (%)	Mean age (y) at onset (range)	Age at onset (median, y)
Dysphagia/Feeding intolerance	49 (37.12)	5.77 (0–24)	3.67
Failure to thrive	36 (27.48)	1.66 (0–10)	0.75
GERD	29 (22.14)	1.47 (0–13)	0.40
Feeding tube placement	20 (15.27)	6.34 (0.13–21)	3.25
Complete reliance on feeding tube	12 (9.16)		
Recurrent vomiting	8 (6.11)	1.46 (0–6)	1
Liver dysfunction	4 (3.10)		
IBD	2 (1.54)		
Other GI symptoms	8 (6.2)		

Dysphagia or feeding intolerance was the most reported manifestation, accounting up to 37.1% of our cohort, with a mean age at onset of 5.7 years (median 3.7). Failure to thrive (according to WHO or CDC growth charts)^1^ was observed in more than a quarter of our patients (27.5%) and was reported at a mean age of 1.7 years, even if half of these patients had growth failure noted within the first year of life (median 0.75 year). GERD was also diagnosed early in life (mean age at onset 1.5 years, median 0.4 years) in 22.1% of our patients. 20 patients (15.3%) required feeding tube placement at a mean age of 6.3 years (median 3.25 years) and 12 (60%) had a complete reliance on gastric feeds (9.2% of the whole cohort). Recurrent vomiting (6.1%), liver dysfunction (3.1%), and inflammatory bowel disease (1.5%) were also reported. Other gastrointestinal abnormalities (e.g., stypsis, recurrent diarrhoea, abdominal pain, vomiting, nausea) were noted in 6.2% of patients (Table 1).

Our series highlights the relevance of GI disorders in patients affected by leukodystrophies. Emerging evidence underscores the intricate interplay between GI disorders and Gut Microbiota (GM), highlighting the bidirectional nature of this relationship, wherein GI disorders can perturb the delicate balance of GM composition (19). Alterations in GM, in turn, have been implicated in influencing the pathophysiology of neurodegenerative diseases (20). These findings underscore the critical importance of understanding and potentially modulating GM in the context of both GI and neurological health, thereby modulating the clinical outcomes (21). However, no studies have been conducted so far on GM and disease outcomes in patients with leukodystrophies.

## 5 Gastrointestinal disorders and gut microbiota

The human GI tract is one of the biggest interfaces between the host and the environment, with symbiotic microorganisms that offer many benefits to the host. The GM composition varies between individuals and evolves through the host’s lifespan, and it is influenced by intrinsic and extrinsic factors (22). Among the major factors able to influence GM composition are the composition of maternal microbiota, maternal health and nutrition status before and during pregnancy, lactation, type of childbirth and diet. Geographic area of residence, antibiotic use, smoking exposure, as well as the health of immune system are also proven to impact GM (23, 24). Diet represents one of the main variables that affect the composition of GM, possibly leading to diversification of the microbial populations. The microbial composition of the small intestine plays an important role in modulating gastrointestinal processes such as secretion and motility and digestive functions, in addition to maintaining a tight communication with the CNS via the microbiota-gut-brain axis (MGBA) (25, 26).

The association between gastrointestinal disorders and microbiota alterations has been analysed in animal models. Kashyap et al. (27) utilized controlled mouse models to investigate the relationship between diet, transit time and GM. They demonstrated changes in gut microbial communities associated with variations in gut transit time by either speeding up or slowing down host gastrointestinal transit, administering polyethylene glycol or loperamide, respectively. These alterations in microbiota returned to normal levels after discontinuing the treatments. In contrast, introducing a diverse fecal microbiota from healthy humans into germ-free mice significantly reduced gastrointestinal transit time and enhanced colonic contractility. The different response depended on the quality and quantity of carbohydrates consumed with diet, as fermentable polysaccharides alter the composition of gut microbiota and the production of metabolites, i.e., short chain fatty acids (SCFAs) (27).

The intricate relationship between GI disorders and the GM is also the focus of several recent clinical studies, that explore the complex interplay between different microbial communities and various GI conditions. Irritable Bowel Syndrome (IBS) is a prevalent functional GI disorder characterized by recurrent abdominal pain and altered bowel function. It represents a good example of GI disorder, given the complex pathogenesis, that potentially involves genetic predisposition, environmental factors, and gut dysbiosis (28, 29). Through metagenomic analyses and 16S rRNA gene sequencing, a dysregulated GM composition has been unveiled in these patients, characterized by alterations in microbial diversity, abundance, and metabolic function (29). Dietary interventions have emerged as promising avenues for modulating gut microbial composition and alleviating IBS symptoms, underscoring the bidirectional relationship between diet, GM, and clinical outcomes (28, 29).

GM has been extensively studied in GERD as well. Indeed, intestinal dysbiosis has been described in cohorts of patients with GERD and seems to be associated not only to the pathogenesis of this condition itself (30), but also to the specific pharmacological treatment to which these patients are subjected (31). A recent review by Kiecka et al. provides an overview of the most important effects of long-term proton pump inhibitors (PPIs) use (32). Among them, gut dysbiosis, probably due to their mechanism of function, is reported. In fact, PPIs exert profound effects on gastric acid secretion, thereby altering the luminal pH and perturbing microbial equilibrium within the GI tract. To support this evidence, probiotic supplementation has emerged as a promising strategy for restoring gut microbial homeostasis and ameliorating adverse sequelae associated with PPI-induced dysbiosis by replenishing beneficial microbial strains and enhancing mucosal barrier function (32). Indeed probiotic strains, such as *Lactobacillus reuteri* (DSM 17938), have appeared promising, showing mitigating efects in children on PPIs therapy. Other interesting strains with potential protective function include *L. rhamnosus* LR06 (DSM 21021) or *L. pentosus* LPS01 (DSM 21980) (32).

Finally, numerous microbial products have been recognized as regulators of GI motility and are implicated in the pathogenesis of colonic motility disorders. These include short-chain fatty acids (SCFAs), bile acids, tryptamine, as well as various gaseous byproducts such as methane, hydrogen sulfide, and hydrogen gas (33, 34).

## 6 Gastrointestinal function and microbiota-gut-brain axis (MGBA): the bidirectional communication

**Figure 1:**
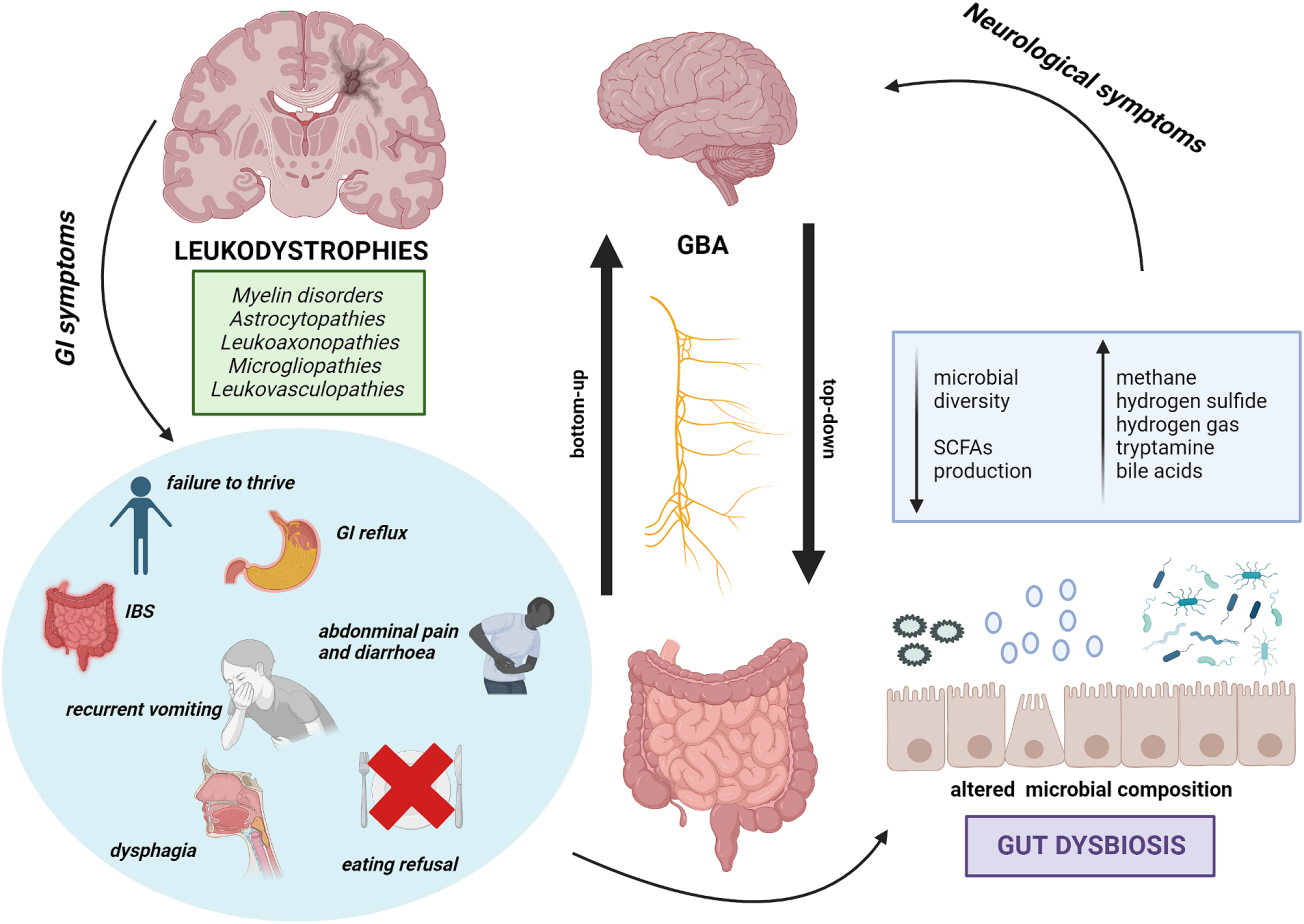
The bidirectional communication between GI disorders in Leukodystrophies and MGBA. Representation of the relationship between gastrointestinal symptoms in leukodystrophies and their potential impact on neurological severity. Key GI symptoms may determine alterations in GM, suggesting a link between dysbiosis and worsening of neurological symptoms. This implies a possible connection between GI issues in leukodystrophies and neurological severity.

The well-known close and bidirectional communication between brain and intestine happens via the microbiota-gut-brain axis (MGBA). GM can influence the systemic health by contributing to the signaling along the GBA, whereas the Central Nervous System (CNS), Enteric Nervous System (ENS), neuroendocrine and neuroimmune pathways are all involved in the bidirectional communication between the CNS and the GI tract (35, 36). Top-down communication refers to the transmission of information from brain-to-gut whereas the bottom-up to the one from gut-to-brain (37) (Figure 1).

### 6.1 The top-down communication

Recently, several studies have highlighted the influence of modulations in the GM on behavior and disease severity in animal models of neurodevelopmental, neurodegenerative, and psychiatric disorders (38, 39). It is fully understood that a communication between GM and CNS does exist, and it is referred to as MGBA, which plays a pivotal role in maintaining homeostasis in the gastrointestinal tract, CNS, and microbial systems. This regulation is achieved through a complex network of chemical transmitters, including endocrine hormones, microbial molecules, and metabolites (40). GM plays an important role in the regulation of neurodevelopmental processes, including blood–brain barrier (BBB) formation and integrity, microglial maturation and function, and myelination, whose disruption could have a role in neurodegenerative diseases (41). According to recent studies, the MGBA is essential for controlling several physiological functions as well as pathophysiologic processes (21). It is now evident that the gut has direct control over the brain, and the brain exerts an effect over the gut functions. Evidence in animal research derives from investigations on infections, antibiotics, and fecal transplants, as well as from germ-free animal models (21). Via the ENS, the Vagus nerve directly regulates different gut processes, many of which have an impact on the GM, gut motility, intestinal permeability, bile and enzyme secretion, mucus production, nutrient absorption, and satiation. In addition, the Vagus nerve regulates inflammation. To maintain equilibrium in the human organism, a balanced and healthy microbiota is crucial. The disruption of eubiosis (i.e the dysbiosis status) causes the loss of homeostasis, richness, and evenness of microbial species, favoring disease onset.

### 6.2 The bottom-up communication

Gut dysbiosis may result in chronic inflammation, which has critical effects on the brain. In fact, it promotes the aggregation of misfolded proteins around neurons at the CNS level, disrupting neuronal function, survival, and hence synaptic integrity. The death of neuronal cells leads to the release of misfolded neurotoxic aggregates, further exacerbating neuroinflammation (42).

Chronic inflammation and oxidative stress determined by gut dysbiosis have been explored in several neurodegenerative disorders, such as Parkinson disease (PD), Alzheimer’s Disease (AD), Multiple sclerosis (MS) and Amyotrophic Lateral Sclerosis (ALS) (43). In PD, gut dysbiosis has been shown to trigger and promote 𝛼-synuclein fibril formation and dissemination, and the transplantation of fecal microbiota from PD patients to 𝛼-synuclein-overexpressing mice worsened inclusion bodies and parkinsonian symptoms compared to mice receiving healthy donor microbiota (40).

Research by Raval et al. (43) suggests a connection between GM dysbiosis and heightened inflammation and intestinal permeability in AD progression. Inflammatory reactions resulting from GM dysbiosis contribute to the breakdown of gut epithelial barriers, facilitating the entry of gut bacteria, fungi, and their products into the brain. Individuals with AD exhibit elevated bacterial levels within the brain compared to those without the condition. This invasion of GM components into the brain may contribute to both peripheral and central innate immune system dysfunction, characteristic of AD pathology (44). Furthermore, products derived from GM, such as lipopolysaccharides (LPS), microbial amyloid, and neurotoxins, have been implicated in neurodegeneration, amyloid-beta aggregation, neurofibrillary tangle formation, and neuroinflammation within the brain (45, 46).

Perturbations in the GM of children affected by MS compared to children without MS (47) and associations between GM and MS activity in children have been demonstrated (48). Studies about transplantation of MS patients’ microbiota into two different animal models of MS have highlighted the importance of interleukin IL10- producing CD4 T cells in the immunomodulatory effects of the GM (49, 50). Furthermore, the presence of specific Gram-positive bacteria in the gastrointestinal tract, which activate Th17 cells, significantly affected the severity of the disease in mice (49). In addition, converging data from germ-free mice and antibiotic preclinical studies have implicated the microbiota in regulating myelin production in mouse prefrontal cortex (50, 51).

The relevance of GM has been also demonstrated in animal models of neurodegenerative disorders which usually have their onset in childhood. For example, autophagic dysfunction and GM dysbiosis have been demonstrated to cause chronic immune activation in the Drosophila model of Gaucher disease, through chronic activation of NF-kB signaling in the Gba1 loss-of-function model. Atilano et al. (52) observed that restoring microbiota or stimulating autophagy to remove immune mediators, rather than administering prolonged immunosuppression, may represent effective therapeutic avenues for GBA1-associated disorders. Kovàcs et al. (53) reported that the GM of mouse models of ceroid lipofuscinosis is altered as compared to wild-type mice. They demonstrated that acidified drinking water markedly changed the GM composition of Cln1 mice, reduced the abundance of the pro-inflammatory microorganisms, determined a decrease in the amount of lysosomal storage material in every brain region examined, reduced astrocytosis in the striatum and somatosensory cortex, attenuated microglial activation in the thalamus, and preserved the ability of Cln1 mice to climb down a vertical pole as quickly and proficiently as wild-type mice (53).

The composition of GM in neurodevelopmental disorders (ND) and its potential impact on brain functions and behaviors is the topic of a recent narrative review (54), which highlighted the role of gut microbes and their metabolites in directly or indirectly influencing brain function. In particular, it was noted that an increase in Clostridium spp. can lead to elevated production of indole, which suppresses the growth of beneficial bacteria like Bifidobacteria and Lactobacilli, ultimately affecting gamma-aminobutyric acid (GABA) levels (55). This mechanism has been associated with occurrences of stereotypies, hypersensitivity, and epilepsy. Furthermore, toxins produced by Clostridia exacerbate inflammatory responses. In other NDs, certain microbial species such as Enterobacteriaceae, Sutturella spp., and Erysipelotrichaceae also contribute to inflammation, leading to alterations in gut permeability and gastrointestinal symptoms (56). Additionally, a high protein diet in ND patients promotes the production of branched chain fatty acids (BCFAs) and propionate (57), with the latter showing behavioral impairment in animal models, suggesting the potential for microbiome-based treatments.

### 6.3 MGBA in leukodystrophies

Composition in GM has been explored in one adult-onset leukoencephalopathy, namely cerebral autosomal dominant arteriopathy with subcortical infarcts and leukoencephalopathy (CADASIL) (58). In the GM from 15 Japanese CADASIL patients, a notable rise in the presence of certain bacteria was observed, including Lachnospira, Odoribacter, Parvimonas, unidentified genera within Barnesiellaceae and Lachnospiraceae families, compared to paired controls. Conversely, there was a significant decrease in the presence of Megasphaera and Acidaminococcus. When comparing CADASIL subgroups, those who had experienced a stroke displayed a significant decrease in Phascolarctobacterium and Paraprevotella. Potential impact of certain genera on C-reactive protein levels was highlighted, as well as their role in stimulating the production of interleukin-10 (IL-10) and transforming growth factor-beta (TGF-β) (58), suggesting that GM composition may not only affect the onset but also the progression of CADASIL.

To the authors’ knowledge, no studies have been conducted to date on MGBA and disease outcomes in leukodystrophies. Expanding upon the work that has been done with CADASIL, it could be worthwhile to investigate the potential effects of MGBA on the phenotype of other leukodystrophies. Indeed, there is often no clear genotype–phenotype association in these diseases, and current research has focused on potential phenotypic modifiers. Given the significant role that GI disorders play in leukodystrophies and the intricate relationships that drive MGBA, unraveling the eventual influence of GM on disease phenotype could mark a significant advancement in comprehending the remarkable phenotypic heterogeneity that has been noted in leukodystrophies.

## 7 Discussion

Several studies highlight the bidirectional link between gastro-intestinal disorders and altered GM, and the existence of a gut-brain axis is nowadays widely accepted. Thus, a deeper understanding of how the gastrointestinal and nervous systems interact together with the GM mediation is needed. Studies on the impact of dysbiosis and MGBA dysfunction in neurological diseases are increasing, especially in the field of neurogenerative disorders. Though, studies on the role of gut-brain axis and microbiota alterations in paediatric-onset neurodegenerative conditions are scarce.

Basing on these assumptions and focusing on leukodystrophies and genetic leukoencephalopathies, which are among the most frequent neurodegenerative disease in children, we may speculate that GI disorders in patients with leukodystrophies may contribute to dysbiosis, leading to altered processes in both the gut and brain, and contributing to neurodegeneration. The loss of blood–brain barrier integrity, which may also be influenced by the GM, promotes the translocation of gut microbes and their metabolites, potentially contributing to inflammation, oxidative stress, pathological protein aggregation, abnormal proteolysis, and neuronal death. These processes are known to play crucial roles in the pathogenesis of various neurodegenerative disorders, including some leukodystrophies (59, 60). Furthermore, considering the essential role of the GM in immune system development and maturation, it is reasonable to suspect its involvement in the pathogenesis of neurodegenerative disorders with a significant inflammatory component (61–64).

To our knowledge, the literature lacks systematic studies investigating the prevalence of GI disorders in patients affected by leukodystrophies. A study conducted by Kay-Rivest et al. (9) reported dysphagia in 7 out of 12 (58%) leukodystrophy patients recruited, with 3 (43%) being completely reliant on a gastric tube. While these results may be slightly biased due to a small sample size, they are consistent with the findings in our cohort. Our results underscore the importance of conducting a comprehensive gastroenterological and nutritional assessment in children affected by white matter disorders. All children with leukodystrophies should have their growth patterns monitored using growth charts, and accurate dietary data are essential for adjusting food intake to promote growth and maintain gut eubiosis. In children affected by neurological impairment (NI) with long-term enteral nutrition, a significant impact on gut microbiota composition was found, which was in turn linked to an aggravation of their nutritional status (65). The significant prevalence of GI symptoms, such as dysphagia and GERD, underscores the need to deepen our understanding of the influence of the gut-brain axis on the clinical phenotype of these individuals. Therefore, prospective studies aimed at analysing the GM in these disorders are crucial, as our understanding of how gut environment affects neurodegenerative disorders may reshape treatment approaches. To this aim, it becomes relevant to identify adequate biomarkers that confirm and measure the impact of dysbiosis and gut-brain axis dysfunction on disease progression and examine the efficacy of innovative treatments targeting the GM, eventually evaluating the potential role of animal models in this process.

Therapies like biotics and faecal transplants offer potential for customized treatments to improve gut health and function, potentially reducing brain inflammation, limiting protein aggregate formation, and slowing disease progression. This shift toward considering the gut-brain connection as a potential treatment may represent a significant departure from conventional methods and holds promise for improving outcomes and quality of life in patients that deal with neurodegenerative diseases.

## Author contributions

YV: Data curation, Investigation, Methodology, Resources, Writing – original draft, Writing – review & editing. FB: Data curation, Investigation, Methodology, Resources, Writing – original draft, Writing – review & editing. VT: Investigation, Methodology, Writing – original draft, Writing – review & editing. MT: Investigation, Methodology, Writing – original draft, Writing – review & editing. CM: Writing – review & editing, Conceptualization. GZ: Writing – review & editing. DT: Conceptualization, Writing – review & editing. EV: Conceptualization, Writing – review & editing.

## Footnotes

^1^ https://www.cdc.gov/growthcharts/clinical_charts.htm
