# Supplementary material for: Microbiota gut-brain axis: implications for pediatric-onset leukodystrophies
Source: Front Nutr. 2024 Jul 12;11:1417981. doi: 10.3389/fnut.2024.1417981 (PMC11272617; doi:10.3389/fnut.2024.1417981)
Supplement: Supplementary file 1 [file Table_1.pdf]

Supplementary Material

LEUKODYSTROPHIES AND GENETIC LEUKOENCEPHALOPATIES	N
**Pelizaeus Merzbacher Disease (PMD)**	19
COL4A1/2 -Related Disorders	17
Alexander Disease (AxD)	15
X-Linked Adrenoleukodystrophy (X-ALD)	15
H-ABC and *TUBB4A*-Related Leukodystrophies	13
POLIII-Related Leukodystrophy	10
Allan-Herndon-Dudley Syndrome (MCT8 Deficiency)	10
Aicardi-Goutiéres Syndrome (AGS)	9
Megalencephalic Leukoencephalopathy with Subcortical Cysts (MLC)	6
Metachromatic Leukodystrophy (MLD)	4
Mitochondrial Leukoencephalopathy	4
Cockayne Syndrome	4
Zellweger Spectrum Disorders	4
Krabbe Disease	3
Canavan Disease	3
L2-OH-Glutaric Aciduria	3
TMEM63A-Related Leukodystrophy	3
Gangliosidoses (Inc. GM1-/GM2-/GM3-Gangliosidosis)	3
Menkes Disease	2
RARS1-Associated Leukoencephalopathy	2
Pelizaeus Merzbacher Like-Disease (PMLD)	2
PYCR2-Associated Leukoencephalopathy	2
Peroxisomal Acyl-CoA Oxidase Deficiency	1
Vanishing White Matter (VWM)	1
Sulfite Oxidase Deficiency	1
RNF157-Related Disease	1
Leukoencephalopathy with Brainstem and Spinal Cord Involvement and Lactate Elevation (LBSL)	1
PIGB-Related Leukoencephalopathy	1
Desmosterolosis	1
Chloride Ion Channel 2-Related Leukoencephalopathy (CLC-2)	1
KARS-Associated Leukoencephalopathy	1
Hypomyelination With Congenital Cataract (HCC)	1
ELOVL1-Related Leukodystrophy	1
AARS1-Related Leukoencephalopathy	1
EIF2AK2-Related Leukodystrophy	1
Lowe Syndrome	1
NA	8

Table 1. Leukodystrophies and genetic leukoencephalopathies referred to C.O.A.L.A Center.
